# Arteriovenous Malformation Involving Digital Nerves in the Hand: Case Report and Review of the Literature

**DOI:** 10.1155/crip/3818896

**Published:** 2025-08-19

**Authors:** Emi Yasuda, Takashi Nuri, Akinori Asaka, Yoshinobu Hirose, Keigo Osuga

**Affiliations:** ^1^Department of Pathology, Osaka Medical and Pharmaceutical University, Takatsuki City, Osaka, Japan; ^2^Department of Plastic Surgery, Osaka Medical and Pharmaceutical University, Takatsuki City, Osaka, Japan; ^3^Department of Diagnostic Radiology, Osaka Medical and Pharmaceutical University, Takatsuki City, Osaka, Japan

**Keywords:** arteriovenous malformation, case report, digital nerve, nerve involvement, neuropathic pain

## Abstract

Intraneural vascular anomalies are rarely encountered specimens as these are not commonly resected. To the best of our knowledge, this is the first report of the histologic findings in an arteriovenous malformation (AVM) within a digital nerve. We report a rare case of an 18-year-old man with a painful mass in the left hand and middle finger who was referred to our hospital for a treatment strategy consultation. According to Schobinger's clinical classification, the patient was diagnosed with Early-Stage 3 AVMs of the left hand and was followed up for approximately 1 year. Due to increasing pain and dark purple discoloration of the finger, the AVM on the left middle finger was removed en bloc, including the skin, subcutaneous fat, and digital nerve, leaving the tendon and the periosteum. Histology revealed the abnormal vasculature of the AVM within the digital nerve and adjacent subcutaneous fibroadipose tissue. Histologically, two patterns of nerve involvement were recognized: arterioles that pushed into the nerve fascicle in a non-destructive manner, compressing the perineurium, and numerous microvessels in the endoneurium surrounded by microvascular proliferation found on the outside of the perineurium. The presence of intraneural abnormal vessels suggested that one cause of AVM pain was neuropathic.

## 1. Introduction

Arteriovenous malformations (AVMs) are congenital vascular lesions composed of arterial and venous structures that bypass the capillary beds. Histologically, AVMs are composed of dysmorphic arteries and veins, as well as vessels with morphological features intermediate between the artery and vein [1]. Foci of microvascular proliferation (MVP) are common in AVM [2]. Compared with other vascular malformations, AVMs are potentially the most dangerous type of vascular malformation clinically because hemodynamic changes due to the large-volume shunting can lead to ischemic necrosis and limb amputation or life-threatening heart failure and are the most difficult to treat [3]. Furthermore, patients with AVMs in the hands and feet had more severe ischemic pain than those with AVMs in other parts of the body [4]. Young children do not usually complain of pain in AVMs affecting the upper limb; however, symptoms, including pain, may develop later on [5]. As patients age, they are more likely to experience pain, which, in the worse cases, can lead to amputation. The development of pain with advancing age may be due to ischemia, thrombosis, calcification, bleeding, and/or trauma, among other causes. However, in most cases, the exact etiology of pain remains little understood. Herein, we report a case of AVM involving a finger with direct involvement of the digital nerve, which to the best of our knowledge is the first such case. This suggests a direct neuropathic origin of pain in a subset of AVMs.

## 2. Case Presentation

**Figure 1:**
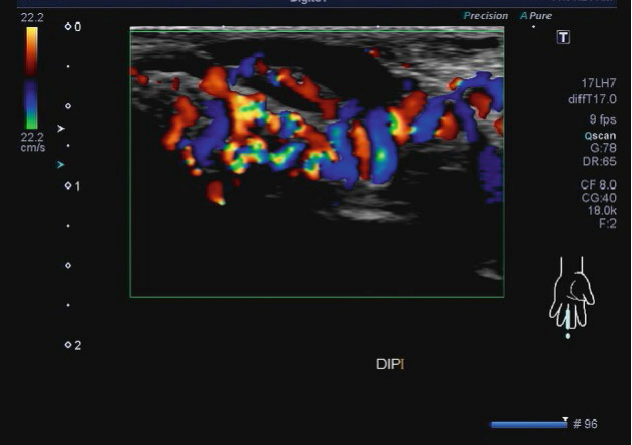
Ultrasound examination image of the ulnar side of the left swollen middle finger. Numerous tortuous dilated vessels with chaotic mosaic-like multidirectional flow were noted from the base of the left middle finger to the distal phalanx.

An 18-year-old man with a painful mass in the hypothenar lesion on his left hand was referred to our hospital for treatment. A pulsatile soft tissue mass (3 cm) was palpable on the left hypothenar prominence, and the left middle finger was swollen with dark purple discoloration and pain with flexion. The pain in the middle finger started when the patient was in junior high school, and the fingertip was particularly painful. The pain worsened after taking a bath or pointing his fingertips down. The patient had a history of childhood asthma. No other vascular anomalies were observed. Ultrasound examination images showed numerous tortuous dilated vessels on both the ulnar and radial sides from the base of the left middle finger to the distal phalanx with turbulent multidirectional flow and shunt waveforms (Figure 1). An arterial component was observed in the left hypothenar lesion; however, the turbulence was minimal. Some turbulent flow was also seen in the thumb.

**Figure 2:**
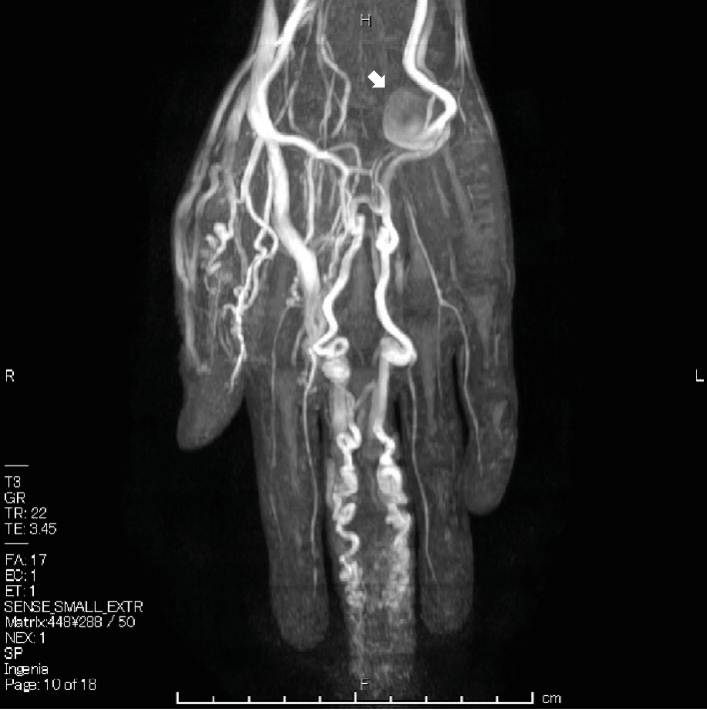
MRA. Tortuous arteries in the left middle finger were shown. A 2.1-cm diameter aneurysm (white arrow) was observed in the hypothenar. MRA: magnetic resonance angiography.

Magnetic resonance angiography (MRA) showed tortuous arteries in the left middle finger, but the structure of the fingertip was difficult to evaluate. An aneurysm was observed in the hypothenar artery (Figure 2).

**Figure 3:**
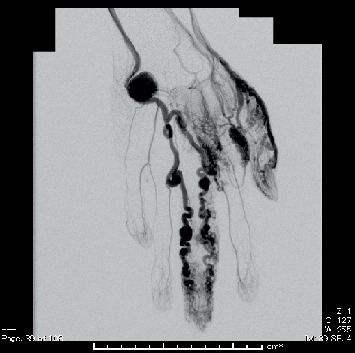
Angiogram of the left brachial artery. An AVM forming a diffuse micro-shunt from the middle to the distal phalanx of the left middle finger was shown, with a dilated meandering of the digital artery of both radial and ulnar sides and some aneurysmal dilation. The hypothenar mass was shown to be a 2.2-cm diameter aneurysm vicinal to the carpal region of the ulnar artery.

According to Schobinger's clinical classification [6], the patient was diagnosed with Early Stage 3 AVM of the left hand because of the presence of pain and was followed up for approximately 1 year. However, the patient experienced increasing pain and finger discoloration (dark purple). There were no signs of hemorrhage, ulceration, necrosis, or inflammation, but the patient opted for treatment while at the university. Angiography was performed to determine the treatment plan. Angiography showed an AVM forming a diffuse microshunt from the middle to the distal phalanx of the left middle finger. Enlarged meandering arteries with aneurysmal dilatation were seen on both (radial and ulnar) sides of the finger. The hypothenar mass was shown to be a 2.2-cm diameter aneurysm vicinal to the carpal region of the ulnar artery (Figure 3).

Since embolization using interventional radiology (IVR) may lead to skin necrosis, radical resection was performed after a discussion between radiologists and plastic surgeons. The AVM of the left middle finger was resected en bloc, including the skin, subcutaneous fat, and digital nerve, leaving the tendon and periosteum, and an extended wrap-around flap from the left big toe was transplanted with vascular and nerve anastomoses. During surgery, when the digital nerve was severed, a pulsatile hemorrhage was observed from the nerve; therefore, the bleeding point had to be cauterized before nerve anastomosis. Simultaneous resection of the aneurysm of the ulnar artery was not performed because of the possible unfavorable effect on the anastomotic vessels. One year and 11 months after the surgery, the patient remained pain-free with no evidence of recurrence. The patient could flex and extend the middle fingers without limitations in daily life. As for the ulnar artery aneurysm, it was under observation.

### 2.1. Histopathological Findings

**Figure 4:**
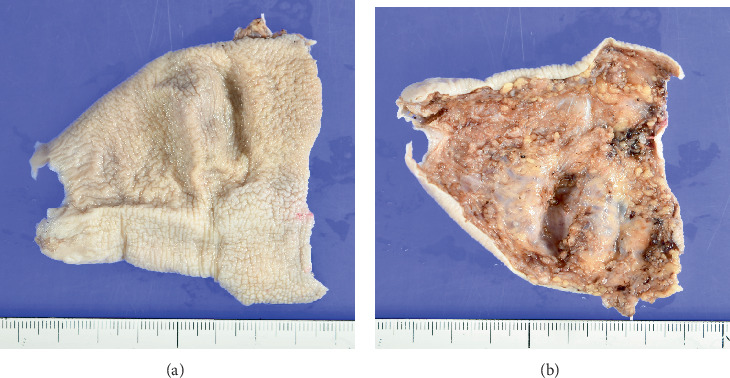
Gross image of the excised specimen of the (a) skin side and (b) dissected surface. The excised specimen was 7.2 × 6.8 cm, with the left side showing the fingertip side and the right side showing the base of the finger.

The excised specimen measured 7.2 × 6.8 cm (Figure 4), and tissue slices were made perpendicular to the long axis from the proximal phalanx to the fingertip. The entire lesions were evaluated via microscopy.

**Figure 5:**
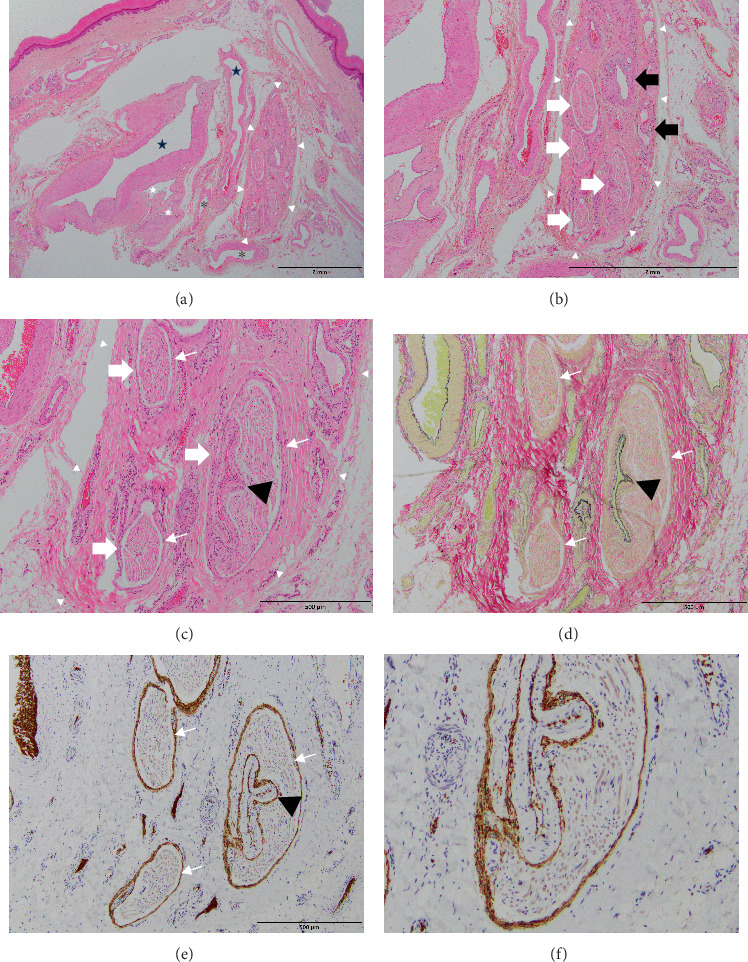
Histological images in the middle phalanx region. (a) Dilated tortuous digital arteries associated with irregular-shaped thick-walled veins were observed in subcutaneous fat, especially on the palmar aspect. Digital nerves accompanying digital arteries and veins were recognized. (b) Abnormal vessels were found within the fibrous epineurium of the digital nerve, accompanying nerve fascicles wrapped in perineurium. (c) Moreover, an abnormal blood vessel seemed to exist in a nerve fascicle wrapped in perineurium. (d) EVG staining revealed the intraneural abnormal vessel had internal elastic lamina-like elastic fibers. (e) IHC for Glut-1 demonstrated the abnormal vessel entering the nerve fascicle in a nondestructive manner within the epineurium and compressing the perineurium. (f) High power view. Black star: artery, white star: vein, asterisk: morphologically intermediate vessels between arteries and veins, white arrowhead: digital nerve, black arrow: epineural abnormal vessel, thick white arrow: nerve fascicles, thin white arrow: perineurium, black arrowhead: intrafascicular abnormal vessel. IHC: immunohistochemical, EVG: Elastica van Gieson.

Dilated tortuous digital arteries up to 0.2 cm in diameter associated with irregularly shaped thick-walled veins were observed in the subcutaneous fat (Figure 5) in all sections from the base to the tip of the finger, especially on the palmar aspect. The following immunohistochemical (IHC) and special stains were performed: Elastica van Gieson (EVG) for vessel walls; CD31, CD34, and podoplanin (D2-40) for endothelial cells; alpha-smooth muscle actin (SMA) for vascular smooth muscle; S-100 for Schwann cells; and EMA and glucose transporter-1 (Glut-1) for perineurium. Glut-1 was also positive in the endothelial cells in the endoneurium. EVG staining revealed focal duplication of the internal elastic lamina and eccentric intimal fibrosis of the arterial components. The venous wall showed focal intimal fibrosis and heterogeneous smooth muscle hyperplasia with sparse elastic fibers. In addition, there were morphologically intermediate vessels between arteries and veins, and normal vessels were present around the lesion. The inflammatory cell infiltration was unremarkable. These findings were consistent with those of AVM. Digital nerves accompanying digital arteries and veins were recognized (Figure 5), and IHC for S-100 protein, EMA, and Glut-1 were useful for the detailed observation of nerve structures. Histologically, nerve fibers consist of the epineurium, perineurium, and endoneurium [7]. Abnormal vessels were observed within the fibrous epineurium of the ulnar digital nerve, accompanied by nerve fascicles wrapped in the perineurium (Figure 5). Moreover, abnormal blood vessels appeared in the nerve fascicle wrapped in the perineurium (Figure 5) and surrounded by S-100-positive Schwann cells. IHC for Glut-1 demonstrated the abnormal vessels entering the nerve fascicle in a nondestructive manner within the epineurium and compressing the perineurium (Figure 5). The intrafascicular abnormal vessels consisted of CD31-positive, CD34-positive, Glut-1-negative, and D2-40-negative endothelial cells and alpha-SMA-positive smooth muscle cells with internal elastic lamina-like elastic fibers (Figure 5), suggesting that they were arteriole-type vessels. The arterioles were recognized in the nerve fascicle in only a few tissue sections. In the sections before and after, they were present outside the nerve fascicle but within the epineurium.

**Figure 6:**
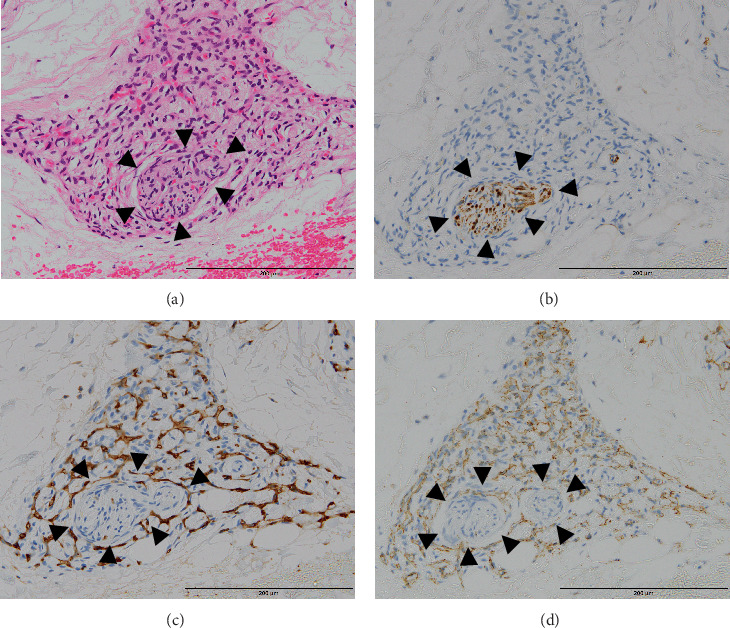
Histological images of the microvascular proliferation (MVP) in the fingertip. (a) MVP surrounded the fine nerve fascicle. (b) Schwann cells in the nerve fascicle were positive for S-100 protein. (c) IHC for CD31 confirmed the presence of microvessels surrounding the nerve fascicle. No obvious vessels were found in the nerve fascicle. (d) Similarly, microvessels were expressed by IHC for alpha-SMA. In this tissue section, the nerve fascicle had been separated into two fascicles. Black arrowhead: nerve fascicle. IHC: immunohistochemical, SMA: smooth muscle actin.

**Figure 7:**
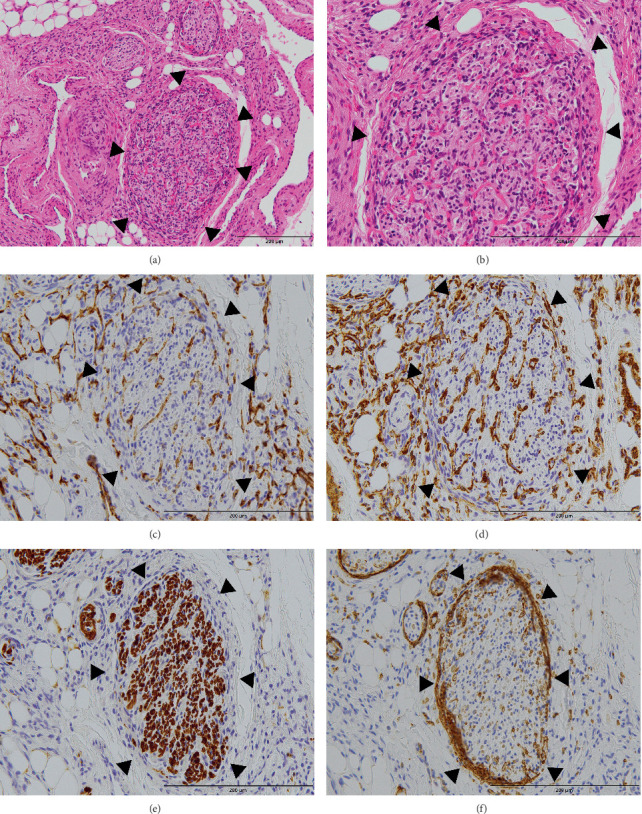
Histological images of microvessels inside the endoneurium in the fingertip. Numerous microvessels resembling MVP were present inside the endoneurium. (a, b) HE stain, (c) CD31, (d) alpha-SMA, (e) S-100 protein, and (f) Glut-1. Black arrowhead: nerve fascicle. MVP: microvascular proliferation, SMA: smooth muscle actin.

In the fingertip, abnormal arteries and veins were recognized in the subcutaneous fat, and highly dilated veins were found immediately below the attenuated epidermis. Furthermore, MVP was observed in the subcutaneous fat and partly in the dermis. MVPs were present only at the fingertip, and several clusters of more than 50 capillaries were observed. Endothelial cell nuclei were plump but not atypical. In this region, many nerves became smaller and consisted of only one fascicle that lacked an epineurium. In some areas, the MVP was present, surrounding the fine nerve fascicle (Figures 6a, 6b, 6c, and 6d); and in a few areas, numerous microvessels resembling the MVP were found, even inside the endoneurium (Figures 7a, 7b, 7c, 7d, 7e, and 7f).

These intraneural microvessels consisted of CD31-positive (Figure 7c), CD34-positive, and D2-40-negative endothelial cells and alpha-SMA-positive pericytes (Figure 7d) with no elastic fibers. Glut-1-positive microvessels were present only at the periphery of the nerve fascicle and most microvessels within the fascicle lacked Glut-1 (Figure 7f), suggesting that they did not have the same properties as the microvessels originally existing in the endoneurium, except at the periphery.

## 3. Discussion

Vascular anomalies are classified as tumors and malformations according to the ISSVA classification [8]. Vascular malformations include capillary, venous, arteriovenous, or mixed malformations, as well as vascular malformation-related syndromes. Although imaging procedures play an important role in the management of intraneural vascular anomalies, microscopic examination is not often performed. The current study shows direct involvement of the digital nerve as a possible cause of pain by microscopic examination.

As vascular anomalies, including vascular tumors and malformations with peripheral nerve involvement, are rarely resected, intraneural vascular anomalies are uncommon specimens and mostly reported in larger nerves, such as the median, ulnar, and sciatic nerves [9–11]. To our knowledge, only eight pediatric and young adult cases with vascular anomalies involving the digital nerve have been reported [12–18], including the present case (Table 1).

Pain was present in all six cases, except for two that were not described. In the present case, the lesion was removed en bloc. Although intraneural involvement was not detected intraoperatively or during gross examination, it was identified upon microscopic examination. In hindsight, persistent bleeding during dissection suggested the presence of high-flow vessels within the nerve. To the best of our knowledge, there have been no reports on the histological details of the relationship between vascular abnormalities and nerves. The final diagnoses of the eight patients included three hemangiomas, two cavernous hemangiomas, one low-flow vascular malformation, one venous malformation, and one AVM. Most of the aforementioned cases were diagnosed before the modern ISSVA classification for vascular anomalies [8] and signed out using obsolete and/or nonspecific diagnostic terminology, such as “hemangioma” or “cavernous hemangioma,” rather than the preferred more-specific modern ISSVA nomenclature. To the best of our knowledge, this is the first report of an AVM with a high-flow vascular malformation involving the digital nerve. Furthermore, our detailed histological study of the relationship between AVM and nerves has not been reported previously, which we believe is a key strength of our study.

Histologically, nerve fibers are composed of the epineurium, perineurium, and endoneurium. The epineurium consists of connective tissue that binds the nerve fascicle. As the nerve branches become smaller and consist of only one fascicle, the epineurium is no longer present. The perineurium wrapping the nerve fascicle consists of concentric layers of flattened cells, forming a blood–nerve barrier. The endoneurium is a compartment that contains axons, Schwann cells, capillaries, and other structures [7]. In the neural component, S-100 protein was positive for Schwann cells, and IHC for Glut-1 identified the perineurium as well as the endothelial cells of microvessels within the endoneurium.

**Figure 8:**
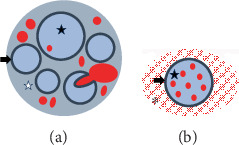
Schematic representation of abnormal intraneural vessels. Excised specimens of the middle finger AVM revealed two patterns of abnormal intraneural vessels histologically. (a) The first pattern. Arterioles were pushing into the nerve fascicle in a non-destructive manner, compressing the perineurium. (b) The second pattern. Numerous microvessels were recognized in the endoneurium surrounded by the MVP found on the outside of the perineurium. White star; epineurium, black arrow; perineurium, black star; endoneurium, asterisk; MVP. AVM: Arteriovenous malformation; MVP: microvascular proliferation.

In the present study, excised specimens of middle finger AVM revealed two patterns of abnormal intraneural vessels histologically: arterioles pushing into the nerve fascicle in a non-destructive manner within the epineurium compressing the perineurium (Figure 8a) and numerous microvessels in the endoneurium surrounded by the MVP found on the outside of the perineurium (Figure 8b).

The first pattern of nerve involvement comprises arterioles pushing into the nerve fascicle in a non-destructive manner, compressing the perineurium, and in some sections, the abnormal arterioles appeared within the endoneurium (Figure 5). It is believed that the arterioles, which originally accompanied the epineurium, were partially curved and entered the nerve fascicle. This abnormality may only be observed in AVMs with highly tortuous arteries. Because the perineurium forms a blood-nerve barrier, pushing into the nerve fascicle in a non-destructive manner is crucial to maintaining homeostasis of the endoneurium. In contrast to AVM, tumor cells were found to invade the innermost endoneurium in the intraneural invasion of malignant tumors [19]; while in AVM, the blood-nerve barrier was preserved.

The second pattern of nerve involvement is associated with MVP. Areas of MVP have been reported mostly in high-flow AVM, indicating a process of angiogenesis [2, 20]. In the present case, MVP was observed around the nerve fascicle, and numerous microvessels were observed within the endoneurium (Figure 7). These endoneurial microvessels were Glut-1-positive only peripherally, suggesting that they have similar characteristics to pre-existing endoneurial vessels; however, most vessels were Glut-1-negative. This means that rather than MVPs around the nerve fascicle invading the endoneurium, there may have been an increase in the existing blood vessels in the endoneurium, and the new vessels may not yet express Glut-1. Numerous microvessels in the endoneurium may not strictly be MVP; however, these microvessels may be affected by growth signals that induce MVP formation. MVP is particularly observed in high-flow vascular malformations; therefore, these vessels may have been observed because of high-flow AVM.

Although ischemia is thought to be the cause of AVM pain [4], its exact causes are currently unknown. Histopathologically, the causes of pain may be difficult to detect; however, if abnormalities or changes in nerve fibers are observed, neuropathic pain may contribute to AVM pain aside from ischemic pain. We demonstrate two new histological patterns of nerve involvement. In the first pattern, even if homeostasis within the endoneurium is maintained, the presence of arterioles with high blood flow in the nerve fascicles may cause pain. In the second pattern, even the mere presence of MVPs surrounding the nerve fascicles could cause pain, as well as the presence of numerous microvessels in the endoneurium, a limited space encircled by the perineurium, could cause pain. Intraneural vascular malformations may contribute to pain, but as the perineurium was preserved and not a destructive nerve invasion, the changes may be reversible. In other words, improving the high-flow abnormality may reduce the number of abnormal vessels in the nerve, leading to a reduction in pain. In the present patient, skin and soft tissue were resected en bloc, but reduction of the arteriovenous shunt by endovascular treatment may also improve pain, as intraneural abnormal vessels may be reduced.

This study has the limitation of having a single case. Further case series focused on the relationship between AVMs and nerves are warranted. However, a detailed histological examination of resected specimens of AVMs may suggest that the new findings seen here may be universal rather than incidental.

In conclusion, this study revealed two patterns of abnormal blood vessels in the nerve: arterioles pushing into the nerve fascicle in a non-destructive manner, and numerous microvessels in the endoneurium, similar to MVPs. Both patterns may have occurred because of the high-flow AVM. Histological evidence of nerve involvement supports the possibility that at least part of AVM pain is neuropathic.

## Figures and Tables

**Table 1:** Review of English literatures on vascular anomalies of the digital nerves.

**Author/year**	**Age/gender**	**Nerve involved**	**Finger number**	**Side**	**History of trauma**	**Pain**	**Macroscopic nerve appearance**	**Macroscopic relation to nerve**	**Diagnosis**
Kon et al. (1981) [12]	8/F	Radial digital + ulnar digital	2	R	NA	NA	Stretched	Intraneural	Extraneural cavernous hemangioma + benign intraneural hemangioma
Nagay et al. (1990) [13]	22/F	Radial digital	2	L	Absent	Present	NA	Intra/perineural	Cavernous hemangioma
Kerimoglu et al. (2006) [14]	9/F	Ulnar digital	4	R	Absent	Present	Expanded	Intraneural	Hemangioma
Galea et al. (2007) [15]	23/M	Ulnar digital	3	L	Absent	Present	NA	NA	Vascular malformation
Duzgun et al. (2013) [16]	12/M	Radial digital	2	R	Absent	Present	Expanded	Intra/perineural	Intraneural hemangioma
Gonzalez Port et al. (2016) [17]	2/M	Median + branch of the nerve innervating thumb and indexfinger	1, 2	R	NA	Present^a^	NA	Extra/intraneural	Venous malformation
Prasad et al. (2016) [18]	25/F	Ulnar digital	NA	R	NA	NA	Enveloped and enlarged	Extra/intraneural	Hemangioma
Present case (2024)	19/M	Ulnar digital	3	L	Absent	Present	Not recognized	Not recognized	Arteriovenous malformation

Abbreviation: NA, not available.

^a^No pain at first examination, but pain 10 years later.

## Data Availability

The data that support the findings of this study are available on request from the corresponding author. The data are not publicly available due to privacy or ethical restrictions.

## Supporting Information

Supporting Information Additional supporting information can be found online in the Supporting Information section. “CARE-checklist-English-2013++.pdf”– Completed CARE checklist in accordance with the CARE guidelines for case reports.
