# Supplementary material for: Arteriovenous Malformation Involving Digital Nerves in the Hand: Case Report and Review of the Literature
Source: Case Rep Pathol. 2025 Aug 19;2025:3818896. doi: 10.1155/crip/3818896 (PMC12380510; doi:10.1155/crip/3818896)
Supplement: Supporting Information — Additional supporting information can be found online in the Supporting Information section. “CARE-checklist-English-2013++.pdf”– Completed CARE checklist in accordance with the CARE guidelines for case reports. [file 3818896.f1.pdf]

CARE
case report guidelines

CARE Checklist of information to include when writing a case report

(cc) BY-NC-ND

Topic	Item	Checklist item description	Reported on Line
Title	1	The diagnosis or intervention of primary focus followed by the words "case report"	Line 3
Key Words	2	2 to 5 key words that identify diagnoses or interventions in this case report, including "case report"	Line 55-56
Abstract (no references)	3a	Introduction: What is unique about this case and what does it add to the scientific literature?	Line 39-40 Line 52-53
	3b	Main symptoms and/or important clinical findings	Line 40-41
	3c	The main diagnoses, therapeutic interventions, and outcomes	Line 42-47
	3d	Conclusion—What is the main "take-away" lesson(s) from this case?	Line 52-53
Introduction	4	One or two paragraphs summarizing why this case is unique (**may include references**)	Line 73-75
Patient Information	5a	De-identified patient specific information.	Line 78-79
	5b	Primary concerns and symptoms of the patient.	Line 78-81
	5c	Medical, family, and psycho-social history including relevant genetic information	Line 83-84
	5d	Relevant past interventions with outcomes	none
Clinical Findings	6	Describe significant physical examination (PE) and important clinical findings.	Line 79-81
Timeline	7	Historical and current information from this episode of care organized as a timeline	Line 78-83
Diagnostic Assessment	8a	Diagnostic testing (such as PE, laboratory testing, imaging, surveys).	Line 84-96 Line 107-112
	8b	Diagnostic challenges (such as access to testing, financial, or cultural)	none
	8c	Diagnosis (including other diagnoses considered)	Line 103-105
	8d	Prognosis (such as staging in oncology) where applicable	not applicable
Therapeutic Intervention	9a	Types of therapeutic intervention (such as pharmacologic, surgical, preventive, self-care)	Line 120-124
	9b	Administration of therapeutic intervention (such as dosage, strength, duration)	Line 120-124
	9c	Changes in therapeutic intervention (with rationale)	Line 120-121
Follow-up and Outcomes	10a	Clinician and patient-assessed outcomes (if available)	Line 128-130
	10b	Important follow-up diagnostic and other test results	Line 132-213
	10c	Intervention adherence and tolerability (How was this assessed?)	unknown
	10d	Adverse and unanticipated events	none
Discussion	11a	A scientific discussion of the strengths AND limitations associated with this case report	Line 240-243 Line 303-306
	11b	Discussion of the relevant medical literature **with references**.	Line 222-243
	11c	The scientific rationale for any conclusions (including assessment of possible causes)	Line 288-298
	11d	The primary "take-away" lessons of this case report (without references) in a one paragraph conclusion	Line 307-311
Patient Perspective	12	The patient should share their perspective in one to two paragraphs on the treatment(s) they received	Line 296-302
Informed Consent	13	Did the patient give informed consent? Please provide if requested	Yes ☑ No ☐
